# Case Report: Epi-endocardial bridges in refractory cavotricuspid isthmus-dependent atrial flutter: technical analysis of epi-endocardial breakthrough

**DOI:** 10.3389/fcvm.2024.1420916

**Published:** 2024-08-08

**Authors:** Andrea Matteucci, Claudio Pandozi, Maurizio Russo, Marco Galeazzi, Enrico Lombardi, Marco Valerio Mariani, Carlo Lavalle, Furio Colivicchi

**Affiliations:** ^1^ Clinical and Rehabilitation Cardiology Division, San Filippo Neri Hospital, Rome, Italy; ^2^ Department of Experimental Medicine, Tor Vergata University, Rome, Italy; ^3^ Biosense Webster, Johnson & Johnson Medical, Rome, Italy; ^4^ Department of Cardiovascular, Respiratory, Nephrological, Anesthesiological and Geriatric Sciences, “Sapienza” University of Rome, Rome, Italy

**Keywords:** cavo-tricuspid isthmus ablation, typical counter-clockwise atrial flutter, epicardial bridge, epiendocardial breakthrough, high-density mapping

## Abstract

**Background:**

Typical isthmus-dependent atrial flutter (AFL) is traditionally treated through radiofrequency (RF) ablation to create a bidirectional conduction block across the cavo-tricuspid isthmus (CTI) in the right atrium. While this approach is successful in many cases, certain anatomical variations can present challenges, making CTI ablation difficult.

**Methods:**

We enrolled four patients with typical counter-clockwise AFL who displayed an epicardial bridge at the CTI. Patients underwent high-resolution mapping of the right atrium and CTI ablation.

**Results:**

Post-mapping identified areas of early focal activation outside the lesion line which suggested the presence of an epi-endocardial bridge with an endocardial breakthrough, confirmed by recording a unipolar rS pattern on electrograms at that site. A stable CTI block was achieved in all patients only after ablation at the site of the epi-endocardial breakthrough.

**Conclusions:**

The presence of an epicardial bridge at the CTI, allowing conduction to persist despite endocardial ablation, should be considered in challenging cases of CTI-dependent AFL. Understanding this phenomenon and utilizing appropriate mapping and ablation techniques are essential for achieving successful and lasting CTI block.

## Introduction

The standard treatment for typical isthmus-dependent atrial flutter (AFL) involves creating a bidirectional conduction block across the right atrial cavotricuspid isthmus (CTI), between the inferior vena cava and the tricuspid valve. Radiofrequency (RF) ablation successfully eliminates the arrhythmia and prevents recurrences by transecting the isthmus in a linear fashion (1). However, achieving a bidirectional CTI block can sometimes be challenging or even impossible. Post-mortem and *in vivo* imaging studies have highlighted anatomical variations in dimensions, endocardial geometry, and muscular architecture across the anatomic landmarks of the CTI (2). These features can pose technical difficulties and contribute to a challenging ablation (3). Examples include a large sub-Eustachian pouch, large pectinates encroaching onto the CTI, and a prominent Eustachian ridge (4). Nevertheless, appropriate procedural approaches can resolve these anatomical issues (4). Another additional factor to consider is the presence of an epicardial bridge, which can create dissociation between the endocardium and epicardium layers of the atrial wall due to the interposed layer of fat. As a result, endocardial RF application may only affect the inner muscular layer, while the outer remains unaffected. Identifying the presence of an epicardial bridge at the level of the CTI level is crucial, especially when it is the cause of intractable CTI AFL (5). In this study, we made a technical analysis reporting four patients with typical counter-clockwise AFL who exhibited an epicardial bridge at the CTI, and in which a stable CTI block was achieved only after ablation at the site of the epi-endocardial breakthrough (EEB).

## Methods and results

### Workflow and electroanatomic mapping

A 7-F decapolar catheter was inserted in the coronary sinus (CS) as local activation time (LAT) reference, regardless of the patient's rhythm (CS pacing or right AFL). A weighted reference across multiple CS electrodes was employed through an advanced reference annotation algorithm. The window of interest (WOI) for local signal annotation was set from +20 ms to +200 ms compared to the reference in the case of CS pacing, or a De Ponti WOI was applied in the case of typical AFL rhythm (6). Then, using a 8-F multipolar star-shaped catheter (40 electrodes) and a 3D mapping system, electroanatomic mapping of the right atrium (RA) was performed during tachycardia or continuous pacing from CS proximal dipole at a frequency of 600 ms. An automatic acquisition was conducted based on the following filters: CL stability ±20 ms, position stability 4–6 mm, LAT stability 4–6 ms, maximum density (≥1 mm), and proximity index impedance-based. The accuracy of the beat acceptance criterion was enhanced thanks to intracardiac pattern matching of the CS's ten unipolar leads morphology, internal point filter at 6–7 mm, map consistency filter to exclude outlier LAT points and the respiration gating compensation. The multipolar catheter signals were filtered at 16–500 Hz in the bipolar electrograms (EGMs) and 2–240 Hz in the unipolar EGMs with an addition mask set at 0.03 mV to exclude signals below this threshold. The quality of the unipolar signals was ensured thanks to the integrated unipolar reference present on the multipolar catheter. This capability allows the operator to better identify near-field EGMs.

### Catheter ablation

Using a point-by-point technique, a set of linear lesions was performed on the CTI with a 3,5 mm tip open irrigated ablation with a contact force sensor. The RF generator was set within a range between 35–45 W, and each lesion was created following a threshold of an index lesion or when a complete positive inversion of the unipolar signal on the ablation catheter occurred, with an inter-lesion distance of a maximum of 6 mm. During the RF applications, the unipolar EGM of the ablation distal electrode was monitored thanks to the 11th electrode of the CS catheter, positioned in the IVC as a unipolar reference. This was fundamental for observing unipolar positive inversion within RF delivery.

### Case 1

**Figure 1:**
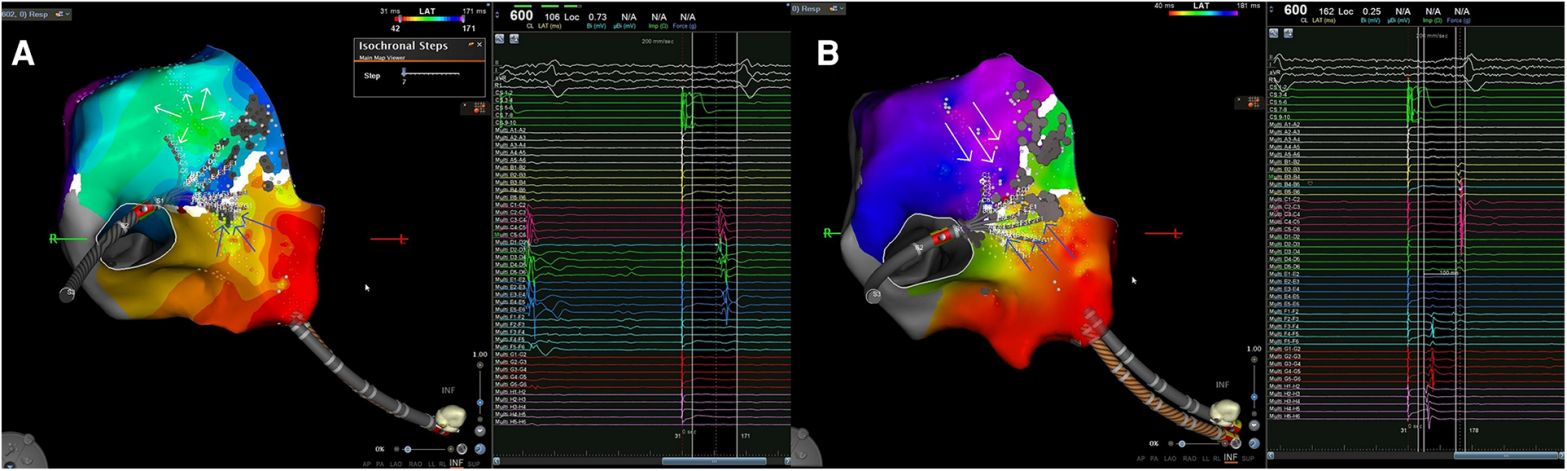
(**A**) Case 1: the isochronal endocardial propagation map demonstrates the epicardial-endocardial breakthrough during fixed pacing from the proximal dipole of CS. The blue arrows indicate the wavefront traveling from the CS towards the CTI ablation line. Subsequently, the first local activation on the other side of the isthmic ablation line is observed 1,3 cm away from it, displaying a focal-type activation (indicated by white arrows in the green zone). Activation proceeds from the site of pseudo-focal activity both laterally and towards the line of the isthmic block (the activation towards the line of the isthmic block is shown also by the recorded EGMs as indicated by the white arrow); (**B**) case 2: in the left picture, the local activation map of right atrium illustrates the CTI block following RF application at the epicardial-endocardial breakthrough; the two wavefronts collide at the CTI level with a time delay of at least 100 ms between the first and second component of the double potential recorded on the isthmus line, as depicted in the EGM on the right. CS, coronary sinus; CTI, cavo-tricuspid isthmus; EGM, intracardiac electrograms; RF, radiofrequency.

A 58-year-old woman with documented typical AFL underwent her first catheter ablation procedure. A 3D electroanatomic map (EAM) of the right atrium (RA) was created using a 3D navigation and mapping technology (Carto 3 system) and a multipolar mapping catheter with spacing electrodes 2-2-2, spine length 1.5 cm (Octaray). Before ablation, a Local Activation Time (LAT) map of RA was generated with pacing from the proximal dipole of the coronary sinus (CS) catheter at a cycle length of 600 ms. The propagation map was automatically acquired using the Confidense module. The first paced map revealed the presence of two wavefronts, traveling the first one downward along the CTI and the lateral wall and the second one upward, towards the right atrial septum; the two wavefronts collided at the anterior rim of the right atrial appendage (RAA). Subsequently, RF ablation was performed along the CTI using the ablation (Thermocool Smarttouch SF) catheter during CS proximal pacing, applying 40 W in power control mode and maintaining an inter lesion distance (ILD) of 6 mm. Once the CTI line was completed, a remap was performed for lesion validation with the mapping catheter to validate the lesions. Although no electrogram (EGM) was detected on the CTI ablation line, an apparently focal potential, preceded by a far-field low voltage electrogram, was observed 70 ms after the stimulus artifact, 1.3 cm inferolaterally respect to the lesion line (Figure 1A and Supplementary Video 1). This phenomenon could be explained by the presence of an epi-endocardial bridge with an endocardial breakthrough (EB) adjacent to the line of block and confirmed by the recording in that site of a rS unipolar pattern on EGMs (7, 8). Complete CTI block was achieved by delivering an additional touch-up RF application at 40 W using an ablation catheter at the site of this apparent focal activation. During the last RF application, the latency between the stimulus artifact and the local activation time increased to a final value of 165 ms.

### Case 2

A 60-year-old man with typical flutter was scheduled for a cavo-tricuspid isthmus ablation. The initial mapping of the RA was performed using the mapping catheter with spacing electrodes 2-5-2; spine length 2 cm (Octaray) with pacing at 600 ms from the proximal dipole of the CS catheter. Similar to the previous case, impulse propagation originated from CS ostium, with one wavefront rising counterclockwise toward the septum, and the second descending along the septum and crossing the CTI. The two wavefronts collided at RAA anterior rim. After identifying the best and shortest pathway of the CTI, RF ablations were delivered using a focal ablation catheter with fixed pacing at 600 ms (see the previous case for ablation settings). Subsequent high-density remapping was performed to validate CTI block, but the delay between the two atrial signals straddling the lesions was still less than 80 ms. The LAT map highlighted early focal activation 1 cm away from the lesions, toward the lateral portion of the isthmus. This indicated the presence of another possible epi-endocardial bridge capable of maintaining conduction through the CTI. We searched for a possible gap in the first lesions set, but we detected the absence of EGMs along the entire line. Consequently, an additional RF pulse was applied on the earliest endocardial activation site while maintaining fixed pacing at 600 ms. This resulted in a further delay between the first and the second atria straddling the line (i.e., Figure 1B). At the end of the RF application, the isthmus was blocked, and the delay between the stimulus from the proximal CS dipole and the atrium on the lateral side of the line reached 167 ms.

### Case 3

**Figure 2:**
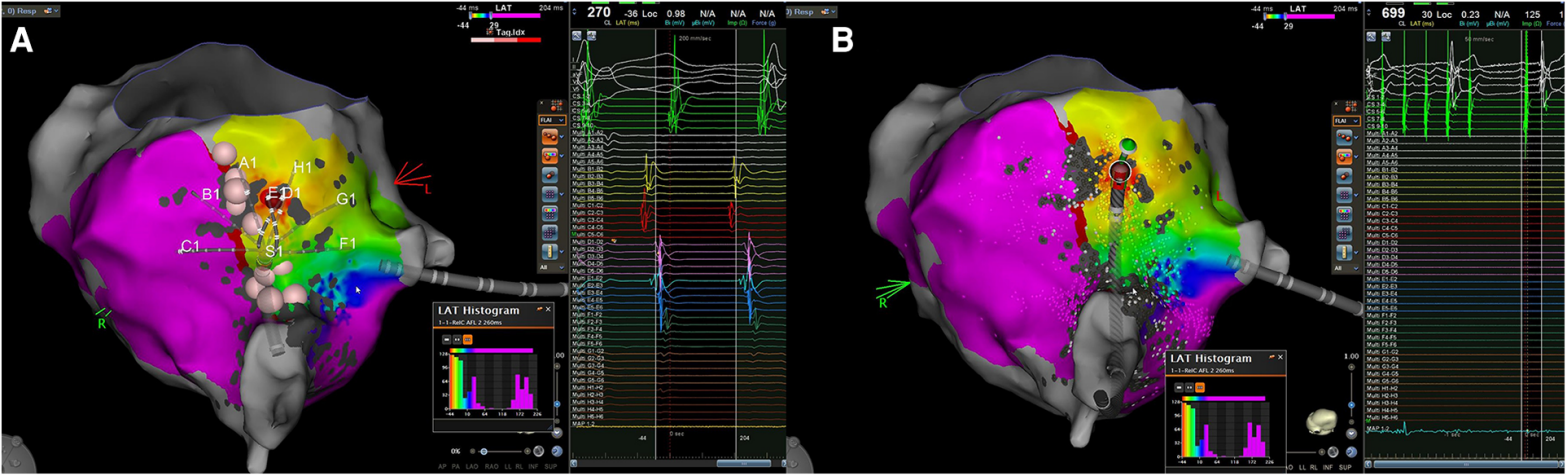
Case 3. (**A**): The local endocardial activation map during typical atrial flutter after performing an ablation line at the CTI level reveals a counterclockwise loop around the TV, with more than 90% of the cycle length occurring within the RA. The propagation map indicates early activation along the CTI at the red spot, located on the septal side of the isthmus, near the TV but far away from the ablation line. Consequently, the typical atrial flutter appears to be sustained by an epi-endocardial breakthrough that can bypass the lesion line; (**B**) in the left picture is shown the application of RF at the supposed endocardial breakthrough resulting in the interruption of the atrial flutter. CTI, cavo-tricuspid isthmus; EGM, intracardiac electrograms; RA, right atrium; SR, sinus rhythm; TV, tricuspid valve.

A 60-year-old man underwent his first catheter ablation procedure for recurrent AFL. ECG recordings showed a recurrence of typical flutter with negative F wave in inferior leads. During the AFL (cycle length 235 ms), 3D electroanatomic mapping was performed using the mapping catheter with spacing electrodes 3-3-3, spine length 2 cm (Octaray). The endocardial propagation map demonstrated counterclockwise wavefront propagation around the TV, with more than 90% of the tachycardia cycle length covered in RA. Subsequently, an ablation line along the CTI was performed with an ablation catheter (Thermocool Smarttouch SF) during arrhythmia, using the SmartAblate system with 40W in power control mode and an ILD of 6 mm, following the point-by-point ablation technique. After completing the ablation line, only a 20 ms increase in the AFL cycle length was observed without interrupting the arrhythmia. Therefore, in the suspicion that the previous flutter had changed the circuit, a second high-density map was made to identify the possible new critical isthmus. Actually, the arrhythmia changed its cycle but maintained the same pathway, as the wavefront propagation was still counterclockwise around the TV and more than 90% of the cycle remained within the RA. However, we noted that, after the wavefront reached the lateral side of the CTI, a focal activation appeared from the septal side beyond the ablation line (Figure 2A). Additionally, no EGMs were present on the ablation line during the remap, suggesting a proper quality of lesions created and the lack of gaps. At the site of the pseudo-focal activity a small area of mesodiastolic potentials with large fragmentation and an rS component on the unipolar signal was found. The flutter stopped after a few seconds of a single-shot RF application (Figure 2B), restoring SR. Finally, a last electroanatomic map was performed with pacing at 600 ms from proximal CS, demonstrating CTI block.

### Case 4

**Figure 3:**
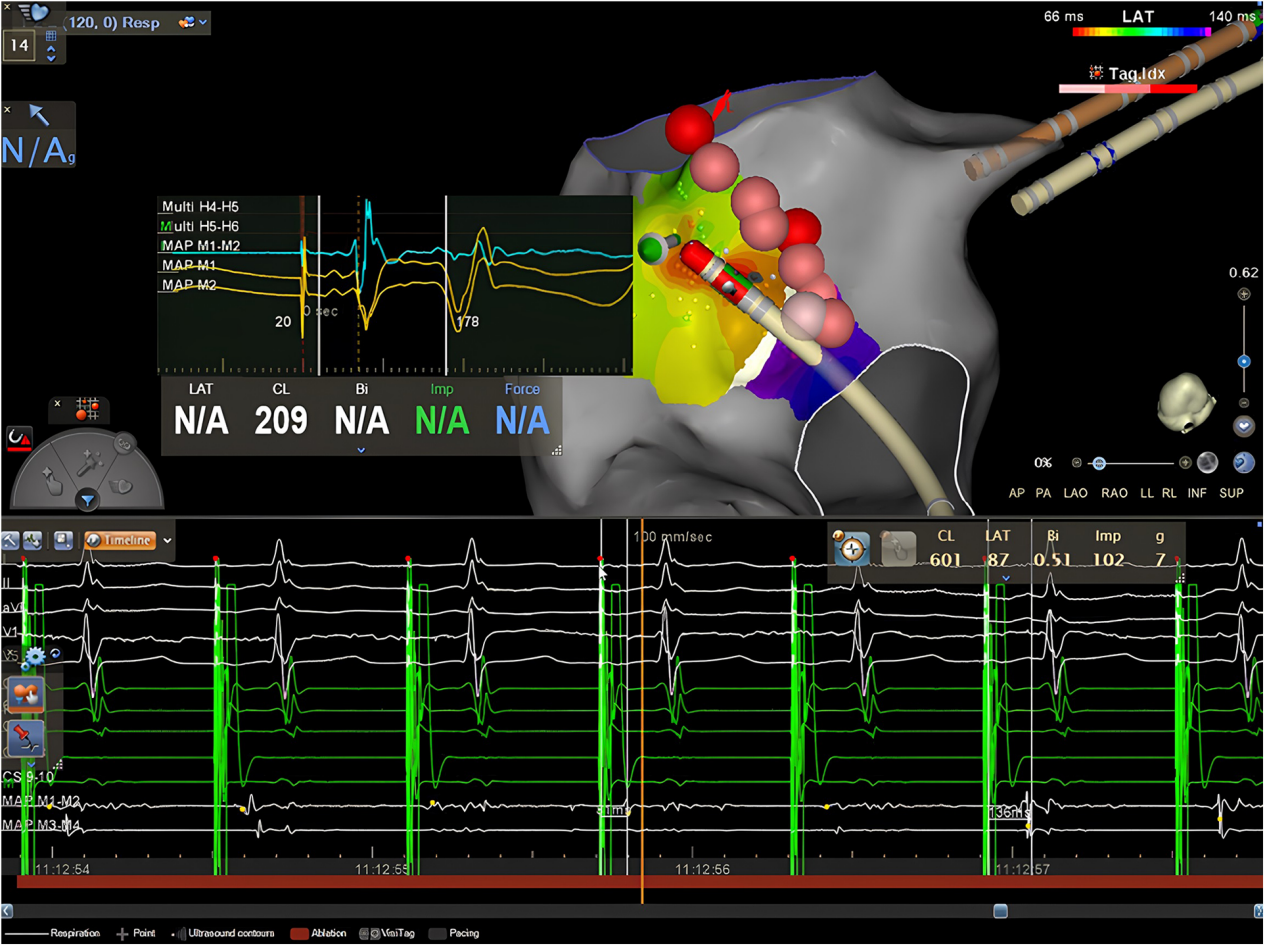
Case 4. Top: isochronal activation map during pacing from the CS shows an apparently focal activation pattern, on the left side of the lesion line, approximately 1,4 cm away from the isthmus. The EGM obtained from the distal dipole of the ablation catheter exhibits early atrial activation with a clear rS pattern preceded by a far-field electrogram (epicardial activation?) both on the bipolar and the unipolar recordings in the middle of the red zone. Bottom: RF delivery at this location increases the stimulus-atrium interval during RF leading finally at the achievement of complete CTI block.

A 63-year-old man with nickel allergy, dyslipidemia, and arterial hypertension underwent his first CTI-dependent AFL ablation with a 3D EAM system. The patient arrived in SR, and a high-density EAM of the RA was performed using a mapping catheter with spacing electrodes 2-2-2; spine length 1.5 cm (Octaray). A shell of the RA was created, and local activation time points were collected while pacing at 600 ms from the proximal dipole of the decapolar catheter placed in the CS. The observed wavefront propagation was identical to that described above in case 1 and 2. Once the optimal pathway of the CTI was identified, RF ablations were delivered using the micro-electrodes catheter (QDOT Micro). A contiguous lesion set was created along the CTI utilizing an ILD ≤6 mm and a point-by-point technique. Subsequently, a remapping of the RA was performed to validate the CTI block revealing that a gap was still present in the middle of the ablation line. Additional RF applications were delivered to perform touch-ups on the isthmus line in an attempt to achieve a complete block. Although the isthmus seemed to be initially blocked, it became unblocked a few minutes later. The remapping of the ablation line showed no EGM potentials detected along the CTI line. However, careful observation of the activation map highlighted the presence of an early “focal” potential beyond the line, 1 cm away on the lateral side of the isthmus (Figure 3, top). Examination of the unipolar lead of the ablation catheter shows a typical rS pattern preceded also in the bipolar electrogram by a far-field slow electrogram (epicardial activation?) suggesting the presence of an epi-endocardial breakthrough (EEB). Additional RF applications with the same ablation settings were delivered on this early focal activation site. During RF ablation, we observed that the isthmus block occurred about 20 s after the start of RF (Figure 3 bottom), indicating a deep conducting channel with a possible multiple endocardial breakthrough. A last high-density remapping confirmed a durable CTI block also after a waiting time of 20 min.

## Discussion

Macro-reentrant isthmus-dependent AFL is sustained by a circuit around the tricuspid annulus and ablation at the level of the CTI creating a bidirectional block is the first-line curative approach with a high success rate (1). Unfortunately, in some cases, it is not easy to achieve a complete bidirectional block, due to the presence of a large sub-Eustachian pouch, a large pectinate muscle, or a prominent Eustachian ridge (4). Papez (9) first reported in 1920 on the complex macroscopic anatomy of the atria muscles showing the presence of overlapping myocardial bundles. His finding was confirmed only years later by the work of Wang (7) and Ho (10) in 1995 and 2002 respectively. Recently, Pambrun (11) described the microtomography imaging and histological analyses of five human donor hearts. These studies revealed a separation between the septopulmonary bundle (epicardial) and the septo-atrial bundle (endocardial) at the level of the left atrial roof due to the intersection of connective or adipose tissue. This separation explains the failure to achieve a transmural lesion during RF applications from the endocardium, leading to the development of epicardial gaps, persistent epicardial conduction, and the inability to create a complete roof line in the dome of the left atrium during ablation of AF or roof-dependent AFL. The increased development of an endo-epi longitudinal dissociation in the atrial musculature, secondary to the increased fibrosis by AF-induced structural remodeling, has been proposed as the electrophysiological substrate of long-standing persistent atrial fibrillation (12). The anatomical architecture of the CT isthmus presents similar aspects to those described for the left atrial roof. Many anatomic studies demonstrated that myocardial bundles are separated by connective tissue both in animals and humans (13). Therefore, the electrical propagation of the impulse through the CTI should be viewed as a 3-dimensional activation process in which electrical wavefronts courses on the other side of the wall. When only endocardial conduction is blocked by non-transmural ablation, the epicardial and endocardial activation conduction might dissociate, and the continuously forward epicardial activation may have a chance to conduct into the endocardium through a distal epicardial-endocardial bridging myocardial fibers. This particular microscopic arrangement may be another important cause for intractable CTI AFL due to the persistence of isthmus epicardial conduction with subsequent endocardial activation through an epicardial-endocardial myocardial connection. This is the case with the patients described in this report.

Our results are in agreement with those reported in the study by Su et al. (5) in which six patients with refractory CTI-dependent AFL showing the presence of an EEB with persistent epicardial isthmus conduction were identified and successfully treated by intensive ablation targeting the central isthmus or the EEB. We have obtained in the same kind of patients a successful ablation with permanent isthmus block by ablation targeted exclusively at the EEB. In our series, epicardial to endocardial breakthroughs during AFL ablation were observed in approximately 6% of cases, involving more than 60 patients, which is in line with the study by Su et al. (5). We found the EEB close to the line of the endocardial conduction block. Immediately after the wavefront reached the line of block, a focal activation, spreading laterally and medially, appeared from the adjacent endocardium on the other side of the line suggesting the presence of a bridging epicardial fiber (Figures 1A, B and Supplementary Video 1). Our results are in agreement also with those reported by Pathic and Coll (14). Showing that there was evidence of a line of endocardial conduction block followed by a focal breakthrough close to this line in a series of patients with CTI-dependent AFL that persisted after the completion of the linear ablation lesion despite the presence of complete endocardial CTI block. The mean distance from the line of endocardial block to the site of focal breakthrough at the posterior RA was 13.6 ± 2.3 mm, similar to that found in our patients 12.1 ± 5 mm. These data indicate that the separation between endocardial and epicardial muscular layers is not continuous and that muscular bridges penetrate through the fibro-adipose tissue connecting epicardial and endocardial muscular sleeves at a variable but relatively short distance. The possible presence of an EB may explain some findings and pose some problems. For example, when bidirectional block across the CTI is difficult to achieve during a conventional ablation procedure without the use of 3D mapping systems and the presence of a sub-Eustachian pouch is suspected, a recommended approach to troubleshooting is to carry out an ablation line more laterally (4). In our experience, by adopting such a strategy, a bidirectional block is achieved often before the new line through the isthmus has been completed. This behavior can be explained either by the muscle bundle hypothesis (15) or by the fact that during the new line realization, at some point, RF delivery may have been applied unknowingly on an EEB of an unrecognized EB. Furthermore, the presence of an EB with an endocardial breakthrough in the posterior pericardial CTI must be differentiated from other situations that may mimic it; for example, limited endocardial mapping after ablation to validate the presence of CTI block may show a pattern of activation during pacing from the CS compatible with CTI conduction although the CTI is blocked. In this situation, there is no apparent isthmus block, but, although the isthmus is blocked, a focal activation pattern may appear at the low right atrium because this region is activated through the posterior portion of the IVC orifice, as demonstrated by Scaglione et al. (16). Differentiation of the two situations can only be achieved by accurate high-density mapping that also includes activation of the IVC as performed in our patients and is of paramount importance from an operative point of view. Indeed, in the case of the presence of an epicardial bridge leading to CTI conduction, ablation at the level of the EEB is required, whereas in the other case, no intervention is necessary because the CTI is effectively blocked. An interesting development for transmural validation could be pulsed field ablation (PFA). While its efficacy has been proven in several studies for the isolation of pulmonary veins, its use in other atrial substrates is still debated. Recent data show its application during persistent atrial fibrillation on the mitral isthmus with results in isolation of the isthmus and good safety profiles, against a low rate of coronary spasm (17). A recent study used PFA on CTI isolation during AF ablation procedure testing the safety of this approach with the use of nitroglycerin to prevent coronary spasms (18). The results indicated that without nitroglycerin, ablation of the CTI led to moderate-to-severe vasospasm. However, with repeated doses of nitroglycerin, severe spasms did not occur, and only mild-to-moderate vasospasms were observed at lower frequencies. Although these findings are promising, further studies are needed to confirm the efficacy of PFA for CTI isolation (19) and to investigate potential interactions with the cardiac conduction system (20).

### Study limitations

This study presents some limitations. It primarily focuses on technical aspects and procedural interventions without extensive exploration of clinical outcomes or patient-reported measures, limiting the comprehensive assessment of the intervention's overall efficacy. In addition, the four cases analyzed may not reflect all anatomic variety, which limits the generalizability of the findings to a broader population. Future prospective studies with larger sample sizes and comprehensive outcome assessments are warranted to address these limitations and provide further insights into the management of CTI-dependent AFL.

## Conclusion

Anatomic studies demonstrate that also CTI consists of discrete muscle bundles often separated by connective tissue. This particular fiber architecture can spare the epicardial layer from the injury of radiofrequency applied from the endocardial side and become the mechanism behind intractable CTI by creating an epicardial bridge capable of supporting conduction across the isthmus. Therefore, the presence of an epicardial bridge as the substrate for intractable CTI-dependent AFL should be suspected in all cases of difficult ablation, particularly when the most common anatomical pitfalls have been ruled out.

## Data Availability

The raw data supporting the conclusions of this article will be made available by the authors, without undue reservation.
